# Perceptions of the causes of eating disorders: a comparison of individuals with and without eating disorders

**DOI:** 10.1186/s40337-015-0069-8

**Published:** 2015-09-15

**Authors:** Elizabeth H. Blodgett Salafia, Maegan E. Jones, Emily C. Haugen, Mallary K. Schaefer

**Affiliations:** Department of Human Development and Family Science, North Dakota State University, 283-H EML Hall, #2615, P.O. Box 6050, Fargo, North Dakota 58108 USA

**Keywords:** Eating disorders, Perceptions, Causes, Media, Psychological and emotional problems, Stigma, Education

## Abstract

**Background:**

In this study, we examined perceptions regarding the causes of eating disorders, both among those with eating disorders as well as those without. By understanding the differences in perceived causes between the two groups, better educational programs for lay people and those suffering from eating disorders can be developed.

**Method:**

This study used open-ended questions to assess the beliefs of 57 individuals with self-reported eating disorders and 220 without. Participants responded to the questions, “What do you think was (were) the cause(s) of your eating disorder?” and “What do you think is (are) the cause(s) of eating disorders?”.

**Results:**

A list of possible codes for the causes of eating disorders was created based on a thorough review of the literature. A manually-generated set of eight codes was then created from individuals' actual responses. Frequencies and chi square analyses demonstrated differences in rates of endorsement between those with eating disorders and those without. Participants with eating disorders most frequently endorsed psychological/emotional and social problems, with genetics/biology and media/culture ideals least endorsed. Participants without eating disorders most frequently endorsed psychological/emotional problems and media/culture ideals, with traumatic life events and sports/health least endorsed. There was a difference between groups in the endorsement of the media as a cause of eating disorders, suggesting that those without eating disorders may overly attribute the media as the main cause while those with eating disorders may not be fully aware of the media’s impact. Additionally, while both groups highly endorsed psychological/emotional problems, there was a noticeable stigma about eating disorders among those without eating disorders.

**Conclusions:**

There were noteworthy differences between samples; such differences suggest that there is a need for more education on the topic of eating disorders. Furthermore, despite empirical support for the effects of genetics, sports, and family factors, these were infrequently endorsed as causes of eating disorders by both groups. Our results suggest that there is a need for more education regarding the factors associated with eating disorders, in order to reduce the stigma surrounding these disorders and to potentially aid the treatment process.

## Background

Eating disorders have increasingly become the focus of research studies due to their prevalence, especially in Western cultures. Of the adolescent and young adult populations in the United States, for example, between .3 and .9 % are diagnosed with anorexia nervosa (AN), between .5 and 5 % with bulimia nervosa (BN), between 1.6 and 3.5 % with binge eating disorder (BED), and about 4.8 % with eating disorder otherwise not specified (EDNOS) [1–4]. According to the fifth edition of the DSM, individuals that do not fit the criteria for AN, BN or BED are diagnosed with sub-threshold or atypical conditions that fit under other specified feeding or eating disorder (OSFED) [5]. Due in part to decreased thresholds for the diagnoses of AN, BN or BED in the DSM-V, rates of OSFED have been found to be lower than previous rates of EDNOS, while the rates of AN, BN or BED have stayed the same or slightly increased [6]. Furthermore, the age at onset is concerning, as most eating disorders originate during adolescence [4]. Despite the potentially serious health consequences that result from disordered eating [7], many in the general public believe that issues with eating are due to personal shortcomings [8, 9]. This creates a foundation of stigma regarding why individuals develop an eating disorder (e.g., to be “skinny”) and the purpose the disorder serves (e.g., to gain control). Such stigma may dishonor the actual experience of those who have lived with an eating disorder, as people could assume eating disorders are self-inflicted. In turn, those developing unhealthy habits may be discouraged from seeking help [10].

Previous research has identified biological, psychological, and sociocultural factors related to the development of eating disorders. However, it is important to explore individual narratives to identify similarities and differences among individuals with and without eating disorders. Obtaining such knowledge can help scholars determine the public’s educational needs and better target missing gaps in their knowledge. More accurate information may reduce stigma regarding eating disorders, which may in turn encourage those experiencing symptoms to seek help sooner, as they may no longer fear the negative feedback from peers and family that such stigma causes.

### Factors that contribute to eating disorders identified by research

Research has identified many risk factors, ranging from individual to sociocultural, that contribute to the development of eating disorders. Based on empirical literature, we present some of the most salient factors below.

### Individual factors

Genetics and biology are individual factors that play a role in the development of eating disorders. Genetic contributions to the development of eating disorders have been suggested by twin studies, with heritability estimates ranging from 0.39 to 0.74, depending on the disorder [11]. Abnormalities in the regulation of certain neurochemicals, such as 5-Hydroxytryptamine (HT) and the serotonin-transporter-linked polymorphic region (5-HTTLPR), have been closely linked with eating disorders [11–13]. Further, recent research has identified mutations on two specific genes that have been associated with increased risk of developing eating disorders in families: estrogen-related receptor α (ESRRA) and histone deacetylase 4 (HDAC4) [14]. In addition, early puberty has also been associated with disordered eating behaviors, potentially due to increases or irregularities in circulating sex hormones, especially estrogen [15, 16].

Body dissatisfaction has been commonly identified as an influential risk factor for eating disorders. Individuals dissatisfied with their bodies are at an increased risk of engaging in disordered eating behaviors such as bingeing and purging in order to gain satisfaction and move closer to the thin ideal [14, 17]. Engaging in dieting behaviors also increases the risk for the occurrence of eating pathology such as binge eating and purging [15, 18].

Researchers have recognized perfectionism as a specific risk factor in the development of eating disorders, as this personality trait may lead to a persistent pursuit of the thin ideal [15, 19, 20]. Perfectionism can also be a maintenance factor for disordered eating since it promotes dieting, bingeing, and purging, and enhances eating disorder symptoms, particularly when combined with low self-esteem [12, 15]. Similarly, research has shown that negative affect in general, such as high levels of stress, guilt, hostility, anger, anxiety, and depressed mood, is associated with increases in eating disorder symptoms [12, 13, 17–21].

Sexual, physical, and emotional abuse have all received empirical support as risk factors for psychiatric difficulties, which can include eating disorders [22]. Specifically, research has shown that sexual abuse can occur in 29 % of individuals with eating disorders, and physical abuse may occur in 57 % of individuals [23, 24]. Additionally, emotional abuse is a significant predictor of eating disorder symptoms among women when other types of abuse are controlled for, suggesting that emotional abuse may be particularly salient [12, 25].

### Sociocultural factors

Many sociocultural factors affect the development of eating disorders. In families, for example, mothers’ and fathers’ own body dissatisfaction and dieting behaviors have been associated with their children’s eating-related attitudes and behaviors [26, 27]. Parental weight-related teasing, negative comments about body shape, pressure to lose weight, and encouragement to diet have also been associated with body dissatisfaction, dieting, disordered eating behaviors, and eating disorders among both females and males [12, 15, 26, 28–31]. Furthermore, parents who engage in high levels of parental control, expressed emotionality, critical comments, hostility, or emotional overinvolvement and negate their child’s emotional needs are more likely to have children who develop eating disorders [12, 32].

Peer influences on the development of eating disorders can also be broken down into a variety of factors. Peer pressure to conform to cultural ideals has been consistently identified as an important factor associated with the development of disordered eating behaviors, especially among adolescents [29, 33]. In particular, girls may learn attitudes and behaviors from their peers, such as the importance of being thin and dieting behaviors, through modeling, teasing, and conversations about body image and eating [12, 33]. Similarly, romantic partners play a significant role in the development of eating disorders through negative comments about appearance and encouragement to lose weight, which can lead to weight concerns, body dissatisfaction, and disordered eating behaviors among both men and women [34, 35].

It is also worth mentioning that eating disorders among athletes are common, as there is a large focus not only on being in shape, but on being the fittest and therefore the “best” [36, 37]. There is an even greater risk of developing an eating disorder with participation in certain competitive sports that focus on leanness, such as gymnastics [38]. Athletes who believe that being leaner will increase their performance are more likely to engage in disordered eating [39]. This belief may be encouraged or reinforced by coaches and instructors, further increasing athletes’ risk for developing disordered habits [40].

Lastly, the media has an influential, if often controversial, role in the development of eating behaviors due its representation of the thin ideal. There is support that, regardless of the level of internalized thin ideal, women who were warned that a thin media image was altered experienced lower body dissatisfaction in comparison to those who were not warned the image was altered [41]. A preference for a thin and virtually unattainable body has been associated with the development of eating disorders, particularly AN [42].

A relatively small number of studies have examined individuals’ perceptions regarding the causes of eating disorders [10, 36, 43–55]. Some studies have solely focused on the perceptions of either the general public [10, 43–47] or those with eating disorders [36, 37, 50–55]. Both types of studies have identified a common set of risk factors, with public perceptions and the perceptions of individuals with eating disorders varying slightly [e.g. 48,49]. Overall, both populations have a basic understanding of what eating disorders are and characteristics of each eating disorder [10, 36, 43–55]. However, despite this knowledge, many adults without eating disorders may be unsympathetic to those suffering from eating disorders, believe that having an eating disorder would not be distressing, and report that eating disorders are not difficult to treat [9].

### Public perceptions of factors that contribute to eating disorders

The studies to date that have focused on identifying public perceptions of the factors associated with the development of eating disorders have surveyed individuals drawn from communities or schools. Typically, these samples have been quite large, numbering over 100 [43, 44] or even several hundred [10, 45, 46], and have included both females and males [10, 43, 44, 46–48]. Despite the importance of large samples, all of these studies have been limited in that the researchers did not ask open-ended questions; rather, participants responded to forced-answer questions where they either had to identify which item was a cause of eating disorders or identify to what degree a particular item was a cause.

The public commonly places blame on individuals with eating disorders, suggesting that they have control over their “self-inflicted” illnesses [48]. Of the individual factors associated with the development of eating disorders, the majority of people who do not have eating disorders identify psychological explanations such as emotional state, personality, and low self-esteem [10, 43, 46–48]. The general public also believes that individuals’ own behaviors and attitudes related to body image such as dieting, a desire to be thin, and body image distortion are important factors in the development of eating disorders [43, 47, 48]. Traumatic events, genetics, and sexual abuse were rarely discussed or, if they were mentioned, rated low on the level of significance in causing eating disorders [10, 47].

Although sociocultural factors are less commonly identified as causal factors of eating disorders among the general public, a few factors have received support. Of all the sociocultural factors, family issues were the factors most often identified [43, 46, 48, 49]. Pressure from friends as well as social isolation and loneliness were also perceived to be factors contributing to eating disorders [46, 47]. In one study, the portrayal of thin women in the media was a highly significant cause endorsed by adult women [45].

### Perceptions of individuals with eating disorders regarding causes

In contrast to studies investigating the perceptions of the general public regarding factors associated with the development of eating disorders, most studies we found that focused on individuals with eating disorders used open-ended measures, either via interview or questionnaires. Despite this, one pitfall of the research to date is that it has often involved relatively small sample sizes, ranging from 15 to 36 [36, 37, 49–51]. Only two studies have included samples over 50 individuals [52, 53]. Additionally, almost all of these studies have focused exclusively on women, with only two including a limited number of men [37, 50]. Furthermore, although research has included assessments of individuals with AN [36, 50, 54] and BN [53, 55] or both [49, 51, 52], studies have failed to examine if differences existed in the perceptions of those with AN versus BN, or include individuals with other eating disorders such as BED, EDNOS, or OSFED.

Similar to public perceptions of causal factors, people with eating disorders also identify individual and sociocultural factors. Individual factors commonly identified among samples of those who were diagnosed with eating disorders include perfectionism, emotional problems or distress, stress, unhappiness with appearance, high expectations of self, and lack of control [36, 48, 50–54]. Behaviors and attitudes related to body image, such as weight loss activities, body image distortion, and a belief that thinness equals happiness, were also frequently identified as factors that related to the development of their disorders [48, 53, 55]. Hereditary factors and sexual abuse were not indicated.

Sociocultural influences identified by individuals with eating disorders included the media, family, peers, and sports. Although rarely mentioned, the media was occasionally identified as playing a role through the importance it places on thinness and self-comparison to the thin ideal [36, 37]. Family factors, in contrast, were often cited and included poor parental care, controlling parents, poor relationship with parents, family tension or high amounts of conflict, critical family environment, emotional abuse, and an emphasis on weight [36, 37, 48–51, 53, 55]. Factors associated with peers and sports were also common and included receiving comments or pressure from friends and coaches about appearance, a need to lose weight for sports performance, and poor relationships with peers [36, 37, 53, 55].

### Comparisons of individuals with and without eating disorders

We could only find two studies that examined the perceptions of both individuals with and without eating disorders. First, Haworth-Hoeppner [49] interviewed 21 women with an eating disorder (either AN or BN) and 11 without, asking open-ended questions about the development of eating disorders. In this study, no comparisons were made across the two groups, likely due to the qualitative nature of the project as well as the small sample size. Second, Holliday and colleagues [48] used larger samples of individuals with and without AN and made comparisons across groups regarding the causes of eating disorders and the most important causes. However, this study was limited in that it did not allow participants to describe their own beliefs. Instead, participants responded to a list of eighteen pre-identified causes of eating disorders, which did not allow for individual perspectives and greater depth into the complexity of eating disorders.

### The present study

With the prevalence of eating disorders and young age of onset, examining people’s perceptions of the factors contributing to eating disorders is important. Such efforts can enhance public education and potentially decrease the stigma surrounding eating disorders. The present study specifically examined the differences between what people with and without eating disorders perceived to be the causes of eating disorders in order to better understand people’s experiences with eating disorders as well as to better educate the larger population. We also examined differences regarding the causes of eating disorders according to type of eating disorder, including AN, BN, both, and other (e.g., BED, EDNOS, or OSFED). This study strengthens existing research by utilizing qualitative, open-ended responses as opposed to forced-answer questionnaires so that participants could identify causes using their own opinions.

## Method

### Participants and procedure

This study was reviewed and approved by the university’s Institutional Review Board. Our sample was recruited from flyers and emails distributed at local universities as well as from flyers distributed to local hospitals and clinics in a medium-sized, Midwestern U.S. city. A secure Internet link was provided, which participants used to indicate consent, provide demographic information, and answer several open-ended questions. All participants were first asked, “Do/did you have an eating disorder?” with the answer choices of “yes, currently,” “yes, in the past,” and “no.” Individuals who answered as having an eating disorder, whether past or current, were asked to specify which eating disorder they had/have and for how long.

The total sample consisted of 277 participants: 57 individuals who had a past or current eating disorder and 220 who did not. Consistent with the ethnic composition of the city, most of the sample identified themselves as White (93 %). There were 234 females (84.5 %) and 43 males (15.5 %). The age range of participants was from 18 to 51 (M = 22.39, SD = 5.77).

#### Sample with eating disorders

Of the 57 individuals who had an eating disorder, 26 had AN (46 %), 12 had BN (21 %), 11 had both AN and BN (19 %), and 8 had another type of eating disorder such as BED or EDNOS/OSFED (14 %). Participants reporting having an eating disorder from between 4 months and 22 years (M = 3.70 years, SD = 4.55 years). Similar to the demographics of the entire sample, 93 % identified as White, and the majority of individuals in this sample were female (96.5 %; *n* = 55). Participants ranged in age from 18 to 47 (M = 23.70, SD = 5.84).

#### Sample without eating disorders

Of the 220 individuals who did not have an eating disorder, 93 % identified as White. In addition, 81 % identified as female (*n* = 179). Participants ranged in age from 18 to 51 (M = 22.05, SD = 5.71). In terms of ethnicity and age, both samples were similar; there were no statistically significant differences between samples (*p* = .80 and *p* = .11, respectively). There was, however, a statistically significant difference in gender (*p* = .01).

#### Survey questions and compensation

After completing a series of demographic questions using the secure Internet link, individuals who had an eating disorder were asked the open-ended question, “What do you think was (were) the cause(s) of your eating disorder?” Individuals who did not have an eating disorder were asked a similar open-ended question, “What do you think is (are) the cause(s) of eating disorders?” These participants were then asked to report why they believed that these were the causes or how they learned about them. All participants were invited to participate in a random drawing for one of four $50 giftcards. Interested individuals were given another secure Internet link to provide their contact information if they wished to enter the drawing; this was done to keep the survey responses anonymous.

## Results

### Coding of participants’ reponses

We initially created a list of possible codes for the causes of eating disorders commonly specified in previous research articles (as identified by overview articles on the risk factors or causes of eating disorders [e.g., 12, 15]). This provided us with a basic framework for content analysis [56]. Next, we manually generated a set of codes from actually reading individuals’ responses to the questions, “What do you think was (were) the cause(s) of your eating disorder?” and “What do you think is (are) the cause(s) of eating disorders?” Thus, we were able to identify a unique but relevant set of eight key themes. The eight themes that emerged from the data were: 1) traumatic life events, 2) family problems, 3) social problems, 4) psychological and emotional problems, 5) genetics and biology, 6) media and culture ideals, 7) sports and health, and 8) body image and eating.

Participants’ responses were then grouped under each of these categories. Many participants identified multiple causes of eating disorders, which were therefore grouped under multiple categories. The responses were coded independently by three research assistants, then checked by an additional research assistant and the first author for consistency. This was done to ensure interrater reliability [56]. When a difference in coding existed, the research team discussed the differences and mutually agreed upon a solution. See Table 1 for sample responses in each category.

**Table 1:** Specific examples of cited causes of eating disorders

Coded Category	Causes
Traumatic life events	Sexual assault, college entry, abuse
Family problems	Comments from family, pressure from parents, need for praise, conversations about weight
Social problems	Bad romantic relationship or break-up, pressure from peers, teasing, social isolation
Psychological and emotional problems	Stress, depression, anxiety, need for control, perfectionism, low self-esteem
Genetics and biology	History of eating disorders in family, chemical imbalance in the brain
Media and culture ideals	Thin ideal images and messages
Sports and health	Gymnastics or dance, health or exercise class, desire to be healthy, lack of knowledge about nutrition
Body image and eating	Drive for thinness, unhappiness with appearance, feeling overweight or unattractive, distorted image

### Frequencies of individuals reporting each cause

#### Sample with eating disorders

A Chi square test for goodness of fit indicated that the participants in this sample showed significantly different rates of endorsement among the causes of eating disorders, χ^2^ (7, *n* = 108) = 41.63, *p* < .05. Specifically, psychological and emotional (*n* = 30) and social problems (*n* = 22) were most frequently endorsed, with the lowest number of endorsements for genetics and biology (*n* = 2) and media and culture ideals (*n* = 5).

Individuals with AN most commonly indicated psychological and emotional problems as the cause (*n* = 13), followed by body image and eating problems (*n* = 9). Individuals with BN reported psychological and emotional (*n* = 8) and social (*n* = 7) as the primary causes. Those with both AN and BN listed all types of problems as causes, so there was not a clear primary cause, although social (*n* = 5) and psychological and emotional problems (*n* = 4) were slightly more frequently endorsed. Finally, those with other eating disorders most frequently cited psychological and emotional problems (*n* = 5) and traumatic life events (*n* = 3). See Table 2 for a complete listing of the frequencies of individuals citing each causal category.

**Table 2:** Frequencies of individuals citing each category for the causes of eating disorders

Category	Frequency according to Eating Disorder				
	Anorexia	Bulimia	Both	Other	No ED
Traumatic life events	5 (19 %)	3 (25 %)	2 (18 %)	3 (38 %)^2^	5 (2 %)
Family problems	7 (27 %)	2 (17 %)	1 (9 %)	1 (13 %)	28 (13 %)
Social problems	8 (31 %)	7 (58 %)^2^	5 (45 %)^1^	2 (25 %)	57 (26 %)
Psychological and emotional problems	13 (50 %)^1^	8 (67 %)^1^	4 (36 %)^2^	5 (63 %)^1^	141 (64 %)^1^
Genetics and biology	0 (0 %)	0 (0 %)	2 (18 %)	0 (0 %)	18 (8 %)
Media and culture ideals	2 (8 %)	0 (0 %)	3 (27 %)	0 (0 %)	104 (47 %)^2^
Sports and health	6 (23 %)	2 (17 %)	2 (18 %)	1 (13 %)	4 (2 %)
Body image and eating	9 (35 %)^2^	1 (8 %)	3 (27 %)	1 (13 %)	57 (26 %)
Total sample size	26	12	11	8	220
Total number of responses given	50	23	22	13	414

*Notes*. Numbers in the table represent how many times each cause was cited by each separate sample. Numbers in parentheses in the table represent the proportion of individuals within each sample reporting that particular cause. Total sample size refers to how many individuals were in each sample. Total number of responses given refers to how many different answers were provided by each sample (i.e., some individuals provided multiple causes of eating disorders).

^1,2^indicate two most frequently endorsed causes for each eating disorder type

#### Sample without eating disorders

A Chi square test for goodness of fit indicated that the participants in this sample showed significantly different rates of endorsement among the causes of eating disorders, χ^2^ (7, *n* = 414) = 326.95, *p* < .05. Specifically, psychological and emotional problems (*n* = 141) and media and culture ideals (*n* = 104) were most frequently endorsed, with the lowest number of endorsements for family problems (*n* = 28), genetics and biology (*n* = 18), traumatic life events (*n* = 5), and sports and health (*n* = 4). Clearly, this sample differed from the sample of individuals with eating disorders in what they viewed as the primary causes. See Table 2 for the frequencies.

#### Differences between samples

Chi square tests for independence indicated that there was not a significant relationship between type of eating disorder (AN, BN, both, or other) and the causes specified. Furthermore, there were no significant relationships among each pairing of eating disorder sub-groups. The lack of statistically significant findings here could be the result of our small sample sizes for each group. See Table 3 for a summary of results from these chi square tests for independence.

**Table 3:** Chi square tests of independence for the relationships between type of eating disorder or no eating disorder and causes specified

Model	X^2^ (df)	Φ
Differences between Samples with Eating Disorders and without Eating Disorders on the Causes Specified	77.96* (7)	.39
(with each eating disorder separated)	98.39* (28)	.43
AN vs. No ED	56.78* (7)	.35
BN vs. No ED	38.64* (7)	.30
Both vs. No ED	23.17* (7)	.23
Other vs. No ED	41.33* (7)	.31
Differences among Samples with Varying Types of Eating Disorders on the Causes Specified	23.04 (21)	.34
AN vs. BN	5.73 (6)	.28
AN vs. Both	8.90 (7)	.26
AN vs. Other	3.66 (6)	.24
BN vs. Both	8.18 (7)	.43
BN vs. Other	1.47 (5)	.20
Both vs. Other	6.01 (7)	.41

*Note*. *p < .05

Of particular noteworthiness, results from a chi square test of independence indicated that there was a significant relationship between eating disorder versus non-eating disorder groups and the causes specified, χ^2^ (7, *n* = 522) = 77.96, *p* < .05, Phi = .39. This suggests that individuals with and without eating disorders had significantly different views regarding the causes of eating disorders, with each group likely to endorse causes at different rates. In conducting follow-up analyses of each cause separately, we found significant differences in the endorsement of family problems (χ^2^ (1, *n* = 39) = 7.41, *p* < .05), social problems (χ^2^ (1, *n* = 79) = 15.51, *p* < .05), psychological and emotional problems (χ^2^ (1, *n* = 171) = 72.05, *p* < .05), genetics and biology (χ^2^ (1, *n* = 20) = 12.80, *p* < .05), media and culture (χ^2^ (1, *n* = 109) = 89.92, *p* < .05), and body image and eating (χ^2^ (1, *n* = 71) = 26.04, *p* < .05) among those with and without eating disorders. More specifically, individuals with eating disorders more often endorsed family problems, and social problems while individuals without eating disorders more often endorsed psychological and emotional problems, genetics and biology, media and culture, and body image and eating.

Additionally, there were significant relations between each individual type of eating disorder versus non-eating disorder and the causes specified. See Table 3 for these results. This suggests, for example, that individuals without eating disorders had different levels of endorsement for each cause than the group of individuals with AN. The same was true for the sub-groups of BN, both, and other, when compared to individuals without eating disorders.

## Discussion

This is the only known study that assessed subjective perceptions of the causes of eating disorders among a relatively large sample of individuals with and without eating disorders. The results support differences between the general public and individuals suffering from eating disorders, which hopefully can be used to provide proper education. Specifically, the general public largely believed that the media causes eating disorders, a perception that is not shared among individuals with an eating disorder. Similarly, sports, body image, and traumatic events were listed less frequently by participants without eating disorders than participants with eating disorders. However, psychological and emotional problems were highly endorsed by all. Together, these findings indicate differences in opinion regarding the causes of eating disorders between those who have an eating disorder and those who do not.

The open-ended questions used in the present study enabled us to gain insight into individuals’ personal opinions regarding factors associated with the development of their disorders, ultimately providing a greater understanding for both clinicians and lay people. Psychological and emotional problems were the most frequently reported causes for those with an eating disorder, supporting the need for greater availability of support systems. In considering the perspectives of individuals who had an eating disorder, it is difficult to know if their perceptions align accurately with the actual causes. However, professionals working with these individuals could help assess the discrepancy between perceived and actual causes. For many postmodern therapists, understanding the perception of the eating disorder from the client perspective and helping him or her make meaning of the experience is more important than determining the actual cause of the disorder [57, 58]. This, therefore, provides reinforcement for the role of psychologists and family therapists within the field of eating disorders, yet many currently lack sufficient training to address eating disorders and instead must refer clients to specialists, who are often expensive and not widely located.

### The role of the media

Our findings revealed a definite contrast between how people with and without eating disorders perceive media as a risk factor for developing an eating disorder. A large percentage of people without eating disorders identified media as a cause (47 %), but only five total participants with eating disorders did. There is a clear separation in the experience of those with eating disorders and with society’s conceptualization of them [36, 37]. Thus, it seems that lay individuals may overemphasize the role of the media as one of the main causes of eating disorders, while those with eating disorders may not be fully aware of the potential impact of the media [50]. Whereas specific media variables such as depiction of the thin ideal and unrealistic body standards may be correlated with eating disorders [42], they do not fully explain disordered behaviors. Our findings should be used to educate consumers of media on the complexity of eating disorders, and as evidence for the need to change the types of messages regarding body image ideals that are currently available in the media.

### Psychological and emotional problems

Psychological and emotional problems were one of the highest named causes of eating disorders by both groups, which is consistent with prior research [43, 46, 48]. However, upon close examination of the data, we noticed a contrast between the written answers of those who had eating disorders and those who did not. More specifically, individuals with eating disorders listed personal reasons, such as “a bad relationship that caused a lot of low self-esteem,” or simple statements such as “stress, depression.” In contrast, there was a negative stigma surrounding some of the answers from participants without eating disorders. These answers included phrases such as “no self-confidence” and “mental disabilities.” This difference is worth noting, because it demonstrates a stigma towards those with eating disorders, which may result in a fear of judgment from others that often prevents those suffering from eating disorders to seek help [59]. Reduction of this stigma through educational programs could encourage individuals who are developing disordered eating habits to speak up, as well as encourage friends and family to begin a non-judgmental, supportive dialogue with individuals about their habits.

### Other factors

Traumatic life events were only listed by 2 % of the non-eating disorder group, versus 23 % of those with eating disorders. This once again emphasizes the need for education geared towards the general public. However, there is also a need for better education for those with eating disorders, as the number of people listing traumatic events was quite low. Many individuals may not make the connection between a traumatic event, such as sexual assault, and the beginning of their disorder, despite empirical support for the effects of abuse [22].

Similar to previous studies, genetics as a cause of disordered eating was only listed by two participants with eating disorders and eight participants without eating disorders [10, 47], making it the least endorsed cause. This indicates a need for the dissemination of information regarding the genetic component of eating disorders, as this could potentially help with the negative stigma surrounding eating disorders [60].

Similarly, and in line with previous studies, only twelve participants with eating disorders and 28 participants without listed family problems as a cause of disordered eating [43, 46, 48, 49]. There are numerous studies, however, that show the impact that mothers, fathers, and siblings can have on the development of disordered eating in an individual (e.g., [26, 27]). If education efforts could help improve understanding of how eating disorders develop within families, parents and siblings can take steps towards preventing the occurrence of these issues and can work towards developing healthier habits for themselves as well.

Sports and health were also listed more frequently as causes by those with eating disorders (19 %), whereas only 2 % of those without eating disorders mentioned them. However, these numbers are both still low. The general public, and specifically coaches, need to be aware of how an intense focus on the body can lead to negative outcomes and strive to support healthy methods of getting and staying in shape.

Body image was listed as a cause of eating disorders by 26 % of participants without an eating disorder, and 25 % of those with experience with disorder eating; these numbers represent a substantial portion of participants. Poor body image often provides a foundation for the development of an eating disorder [15, 17], and understanding what issues underlie an eating disorder can help not only those struggling to recover, but those trying to assist them.

Another highly-endorsed cause of eating disorders was social problems, as 26 % of those without eating disorders and 39 % of those with eating disorders listed them. While these numbers are considerably higher than other groups, only one fourth of those without eating disorders acknowledged social problems as a cause, while a much larger number of those with eating disorders indicated social problems as a cause. However, many individuals may not realize the effect that external events can have on their internal belief systems, once again indicating the need to incorporate this finding into general education, as well as into the treatment process as a way of lessening the blame that those suffering may place on themselves.

### Summary of findings

This study provides insight into the educational resources needed to inform the lay audience regarding eating disorders as well as some factors to consider in the education or prevention of eating disorders among those affected. There is a clear difference between perceived causes of eating disorders from those who have experienced them and those who have not. Those who had not struggled with an eating disorder were more likely to believe that media and cultural ideals influenced eating disorders. For those who had lived with an eating disorder, this was one of the least likely perceived causes. Social problems, in contrast, were frequently listed by participants with eating disorders and less frequently listed by participants without. Genetics and traumatic events were listed most infrequently by both groups, and there were also relatively low levels of endorsement for traumatic life events, sports and health, and family problems among both groups. Both groups listed body image as a fairly frequent cause, and although both groups highly endorsed psychological and emotional problems as causes, there was a clear negative stigma surrounding psychological and emotional problems when listed by non-disordered participants. Improved educational programs should seek to give those who are uninformed a greater understanding of how psychological, social, and relational factors influence those with eating disorders. Increased opportunities for those who have lived with eating disorders to share their stories and perspectives are also needed. With the opportunity to provide first-hand knowledge, these individuals can be an excellent asset for researchers, professionals, and lay people.

### Limitations

Our sample was a relatively homogenous group in terms of gender and ethnicity, so separate analyses could not be conducted examining differences among men and women or among various ethnic groups. Thus, care should be taken when generalizing the results to males and non-white individuals. Furthermore, in order to utilize open-ended questions, no measurement scales were used to determine eating disorder pathology. Therefore, eating disorder status was determined solely by self-report and may not be clinically accurate. In retrospect, it may have been useful to at least provide participants with a self-report survey to assess their eating disorder symptomatology. However, we do note that our sample was recruited not only from local universities but directly from hospitals and clinics that included eating disorder treatment facilities. As a result, we hope that participants were able to appropriately reflect on the nature of their symptomatology. Further, our type of questioning allowed for only two groups of samples, those with eating disorders and those without; individuals who have subclinical symptoms or undiagnosed eating disorders may have been inaccurately placed in the category of non-eating disorder due to their own assessment. Similarly, those who identified themselves as having an eating disorder may have been self-diagnosed, and therefore may not technically meet clinical standards for a disorder.

Additionally, two different questions were asked of participants. Specifically, we asked participants with an eating disorder: “What do you think was (were) the cause(s) of your eating disorder?”, and we asked participants without an eating disorder: “What do you think is (are) the cause(s) of eating disorders?” This allows individuals to add a personal dimension to their analysis of the causes of eating disorders. As such, they may believe that the cause of their disorder is very different than the cause of someone else’s disorder. Similarly, individuals with an eating disorder may have focused more on life events or recent triggers without a reflection on more general risk factors.

Lastly, because this study was completed online, it could be considered relatively impersonal, whereas in-person interviews would have most likely been more in depth. However, because the main interest of the study was to examine participants’ instinctive reactions to eating disorders, the completely anonymous online survey was the most beneficial means of execution.

## Conclusions

Despite limitations, this study contributes to the field in a variety of ways. The sample size of those with eating disorders (*n* = 57) is somewhat larger than samples currently in the literature. Furthermore, while many studies focus only on AN or BN, this study included those with self-reported AN, BN, BED and EDNOS/OSFED, allowing for more inclusive results. It also allowed us to separately assess perceived causes of eating disorders according to the type of eating disorder. For example, individuals with AN most frequently indicated psychological and emotional problems as well as body image and eating problems; individuals with BN often reported psychological and emotional problems as well as social problems; individuals with both AN and BN listed all types of problems; and individuals with BED, EDNOS, or OSFED primarily cited psychological and emotional problems as well as traumatic life events. Although these differences in perceptions were not statistically significant, it may suggest that each type of disorder is unique, with potentially unique causes attributed to the disorder. Future research should continue to examine these differences, and education should focus on the unique nature of each type of eating disorder.

The use of an open-ended qualitative assessment allowed for a complete picture of individuals’ perceptions of the causes of eating disorders. It also allowed individuals to write about more than one perceived cause of the disorders, which is not always possible with close-ended questions with limited answer options. An additional strength of this study is that it contributes to the relatively small pool of current literature discussing perceptions of eating disorders. Within this limited research, most examine perceptions of the general public or perceptions of those with eating disorders separately. Our study is also one of very few studies to examine differences between these two groups.

Overall, it appears that all individuals would benefit from learning more about eating disorders and their causes. Knowing this could be particularly helpful for individuals going through eating disorder treatment, especially for therapists to use when educating those close to someone struggling with an eating disorder. This could help facilitate greater support and connection between family members and friends, and help to end the stigma surrounding these problems and allow those in trouble to seek help.
